# Supplementary material for: No effect of monetary reward in a visual working memory task
Source: PLoS One. 2023 Jan 17;18(1):e0280257. doi: 10.1371/journal.pone.0280257 (PMC9844926; doi:10.1371/journal.pone.0280257)
Supplement: S1 Appendix — (DOCX) [file pone.0280257.s001.docx]

**S1 APPENDIX**

**No effect of monetary reward in a visual working memory task**

Ronald van den Berg, Qijia Zou, Yuhang Li, Wei Ji Ma

**Scoring functions**

In all three experiments, subjects received points on each trial based on the accuracy of their estimate. In Experiment 1, errors were mapped to scores through the function $s=\left[10\cdot e^{-\frac{\varepsilon^{2}}{800}}\right]$, where *ε* is the error in degrees and [.] indicates rounding to the nearest integer (Fig A1, black). In Experiments 2 and 3, the function were $s=10-\left[\frac{\left|\varepsilon\right|}{5}\right]$ and $s=10-\left[\frac{\left|\varepsilon\right|}{4}\right]$, respectively, giving highly similar mappings as in Experiment 1 (Fig A1, red and green).

**Questionnaire items in Experiment 2**

Subjects in Experiment 2 filled out a questionnaire with the following items from the Intrinsic Motivation Inventory [33,34]:

- Interest/Enjoyment
  - I enjoyed doing this activity very much
  - This activity was fun to do.
  - I thought this was a boring activity. (R)
  - This activity did not hold my attention at all. (R)
  - I would describe this activity as very interesting.
  - I thought this activity was quite enjoyable.
  - While I was doing this activity, I was thinking about how much I enjoyed it.
- Perceived Competence
  - I think I am pretty good at this activity.
  - I think I did pretty well at this activity, compared to other students.
  - After working at this activity for a while, I felt pretty competent.
  - I am satisfied with my performance at this task.
  - I was pretty skilled at this activity.
  - This was an activity that I couldn’t do very well. (R)
- Perceived Choice
  - I believe I had some choice about doing this activity.
  - I felt like it was not my own choice to do this task. (R)
  - I didn’t really have a choice about doing this task. (R)
  - I felt like I had to do this. (R)
  - I did this activity because I had no choice. (R)
  - I did this activity because I wanted to.
  - I did this activity because I had to. (R)

Subjects rated these items on a Likert scale from 1 to 7. Scores on items indicated with an (R) were reversed before entering them into the analysis.
