# Supplementary material for: No effect of monetary reward in a visual working memory task
Source: PLoS One. 2023 Jan 17;18(1):e0280257. doi: 10.1371/journal.pone.0280257 (PMC9844926; doi:10.1371/journal.pone.0280257)
Supplement: Attachment — Submitted filename: rebuttal_20220124.pdf [file pone.0280257.s002.pdf]

RESPONSE LETTER TO EDITOR AND REVIEWERS

EDITOR COMMENTS

Dear dr. van den Berg, dear Ronald,

Thank you for submitting your manuscript to PLOS ONE. I have received two very thorough reviews of your paper, which are appended below. As you will see from these, there is a bit of work to be done to meet the journal's publication criteria.

The most important issue that will need to be addressed is the reward manipulation, which is called into question by reviewer 2 for being a "static' between-subjects manipulation. To properly support the conclusions of the paper, a new experiment is suggested in which reward is varied from trial to trial, within subjects. This seems a good approach to me as well. The alternative route of adjusting the current inferences and conclusions on the basis of the existing manipulation seems much more problematic to me.

The reviewers have provided a range of further comments and suggestions, which will also need to be addressed; in particular the "intermediate" points raised by reviewer 2. I expect that this can all be done via textual modifications, however.

We thank the editor and the two reviewers (and the anonymous graduate student who assisted Reviewer 1) for their constructive and thorough comments, which have helped improve the manuscript.

The largest change in the revised manuscript is that we have added the suggested experiment in which reward is manipulated on a trial-by-trial, within-subject basis.

Please find our detailed responses to all Reviewer comments below.

Thank you for considering our manuscript for publication in PLOS One.

REVIEWER #1

Van den Berg and colleagues describe two experiments in which they manipulated the amount of a monetary bonus participants could receive for excellent performance on a delayed estimation task requiring participants to remember orientations. They showed that the amount of this bonus did not affect delayed estimation performance; nor did the amount of the bonus affect other motivational factors measured via questionnaire.

I reviewed this paper in collaboration with a graduate student familiar with delayed estimation who prefers to remain anonymous. We discussed the paper together and agreed on the content of this review. Overall, we think this paper will make a fine contribution to understanding of visual memory. We found the paper to be appropriately concise and well-written. The data analysis

described was appropriate, and so was the data archiving. We agreed that it will be useful for researchers focusing on visual memory to learn that external bonuses do not measurably affect delayed estimation performance. The methods the authors devised for testing this using MTurk were rigorous; in fact, we thought they reflected very conscientious use of online testing. Their procedure included appropriate checks for understanding and engagement from online participants. I particularly liked their method of rewarding participants based on their performance on three random trials. I have used reward manipulations before that summed small rewards per trial (e.g., Morey et al., 2011, Attention, Perception, and Psychophysics), and these could possibly fail to motivate participants optimally, because each trial is worth so little (though note we did find that performance shifted with rewards, but in our paradigm participants shifted between two concurrent tasks based on the reward scheme, which seems quite different). In van den Berg et al.'s version, any trial could have been one that determined the bonus, which seems like a strong way to motivate participants performing many trials. We also agreed that it was interesting and worthwhile to read authors admittedly disconfirming their initial hypothesis. This was expressed in an appropriately measured way.

> Thank you for these positive comments!
>
> It’s interesting that you found flexibility in resource allocation between tasks (despite the small trial-by-trial rewards) – we now refer to this finding in the introduction.

We have a few minor suggestions intended to maximize the readability of the paper:

1) In the procedure, consider explicitly stating that only one of the orientations was tested per trial. This is implied by the figure already, but some paradigms include multiple tests.

> We now mention this explicitly.

2) In Figure 2, using N to denote set sizes is potentially confusing.

> Fixed – we now write it out as “set size”

3) Consider providing a bit more detail about the Intrinsic Motivation Inventory in the Experiment 2 Method.

We have added a little more detail to Methods and included an overview of all questionnaire items in the Appendix.

4) Consider specifying what the key features of "gamifying" a task are (p. 9).

We now mention a few of the key concepts of gamification in that sentence.

Sincerely,
Candice Morey (I always sign reviews) with the collaboration of an anonymous graduate student (who understandably prefers not to sign)

REVIEWER #2

van den Berg et al. present two behavioural experiments, which aim tested the hypothesis that greater monetary incentives improve visual working memory (VWM) performance. In both studies, the authors did not find evidence that extrinsic monetary reward influenced VWM performance. The authors interpret these results as suggesting that resource allocation in VWM is insensitive to monetary reward. While the studies have a pertinent theoretical question, the experimental design does not provide a sufficient test of the experimental aims and the authors' interpretations go behind what can be inferred from these results.

Major concerns:
1. The reward manipulation was insufficient to test the hypothesis.
The studies presented by van den Berg et al. used a between-subjects design in which different groups of participants could earn different amount of total monetary reward for their performance. Reward was fixed within each session and three trials were randomly selected at the end to determine what percentage of the overall bonus would be awarded.
This is a rather unusual reward manipulation. The vast majority of studies investigating motivation-cognition interactions use within-subjects designs, which vary reward trial-to-trial (e.g. Chiew & Braver, 2016; Engelmann et al., 2009; Etzel et al. 2016; Hall-McMaster et al., 2019; Hippman et al., 2019; Kleinsorge & Rinkenaeur, 2012; Locke & Braver, 2008; Poh et al., 2019; Shen & Chun, 2011). An important reason for doing so is that effects of monetary reward on cognition are considered to be relative rather than absolute. Changes in reward prospect induce changes in motivation that affect cognitive processing. Such effects would not be captured in the current design in which reward prospect is static within each subject. Thus, monetary reward might increase VWM resource allocation but this might not be detectable in the between-subject task used here.

Action point: To test the hypothesis that monetary reward increases VWM resource allocation, the authors should run a within-subject online experiment in which extrinsic reward is varied from trial-to-trial. For example, a high or low reward cue could be presented prior to gabor presentation, indicating the maximum number of points that could be obtained on that trial (e.g. 100 for high reward vs 10 for low reward). The authors could then compare circular variance for high and low reward trials as a function of set size. Under the hypothesis that reward increases

VWM resource allocation, one might predict lower circular variance on high reward trials (i.e. a main effect of reward) and an interaction between reward condition and set size, in which the difference in circular variance between high and low reward conditions is lower for larger set sizes. The authors would need to base the final payment on the total number of points in such a design, as selecting a random three trials could undermine subject's efforts to calibrate resource allocation depending on the reward condition.

We thank the Reviewer for this excellent suggestion. We have added a third online experiment (201 subjects) in which the amount of obtainable reward was varied from trial to trial. We did this by adding a "score multiplier" that could take values 1x, 2x, and 4x and which randomly varied between trials. The number of points obtained on a trial – and thus the expected monetary reward – was multiplied by this multiplier.

We tested two groups of subjects that differed in how the reward was calculated. Just as in the first two experiments, we found evidence in favor of *absence* of an effect of reward on encoding precision.

2. The conclusions go beyond what can be inferred from the data.

Given the point above a between-subject design not being suited to detect an effect of monetary reward, the author's conclusions appear to be overstated. Throughout the manuscript, the authors interpret their results as suggesting that VWM resource allocation is independent of monetary reward (lines 23, 73, 210). As outlined above, this might not be the case and caution must be exercised until more appropriate within-subjects experiment/s are run. The most we can conclude is that these results provide evidence against the hypothesis that differences in absolute monetary reward between-subjects influence VWM resource allocation.

Action point: The authors should temper their conclusions at these critical lines (lines 23, 73, 210) to acknowledge their design examines absolute differences in monetary reward between-subjects and that this does not necessarily suggest monetary reward more broadly is independent of working memory allocation. Any conclusions beyond this will depend on the results of within-subject experiment/s.

We believe that this concern was addressed by adding a 3rd experiment, which has strengthened the support of our original conclusions. If the Reviewer believes that the conclusions are still formulated in an overly general way, we'd be happy to consider any suggestions for revision.

Intermediate concerns:
1. The specific behavioural marker of increased VWM allocation is unclear.
The authors distinguish between two forms of flexibility in VWM based on their resource-

rational account of VWM (van den Berg & Ma, 2018). The first is items can be encoded at different strengths based on their importance (e.g. probe frequency and reward associated with the item). The second is the total amount of cognitive resource devoted to VWM can be adjusted depending on the task importance (e.g. the reward at stake). The experiments in this manuscript focus on this second form of flexibility.

Action point: The authors need to make it clear what this second form of flexibility means in concrete behavioural terms. How would an increase in total resource allocation be observed in behaviour? Would this be observed as lower circular variance under higher reward, as interaction between reward and set size on circular variance or both? Put another way, it would be helpful to add concrete behavioural predictions to the manuscript (e.g. at line 71).

To give the reader a better idea of the predictions, we have added results from a simulation analysis in the Appendix. In the analysis, we simulated responses from the resource-rational model of VWM in a reward-manipulation experiment similar to those used in the present paper. The predictions show clear main effects of both set size and reward level on the circular variance of the estimation error, as well as an interaction effect, and stand in clear contrast to the empirical figures presented in the main paper.

2. The conclusion about intrinsic motivation influence on VWM allocation requires clarification/revision.

In their second experiment, the authors collected questionnaire data about subjects' intrinsic motivation. Their first analysis tested whether extrinsic reward condition influenced subjects' scores on the three intrinsic motivation subscales. The authors did not find evidence that this was case. Based on this observed independence between extrinsic reward and intrinsic motivation, the authors conclude that this '… leaves open the possibility that VWM resource allocation may be sensitive to manipulations of intrinsic motivation.' (lines 213-214).

However, the second analysis of intrinsic motivation involved a median split of subjects into low and high motivation groups for each subscale. The authors then examined the circular variance as a function of set size, separately for low and high motivation groups. Results showed evidence towards the null hypothesis that VWM performance did not differ as a function of high or low intrinsic motivation scores for any of the three subscales. Given the evidence that intrinsic motivation did not relate to VWM performance, it is unclear how the authors arrive at the conclusion that manipulations in intrinsic motivation might alter VWM resource allocation.

Action point: The authors should revise or remove this conclusion about intrinsic motivation at lines 213-214. It is true that intrinsic motivation was not manipulated in this experiment. If this is the distinction the authors are wanting to convey (i.e. between extrinsic motivation being manipulated and intrinsic motivation not being manipulated), then this needs to be made clear. E.g. 'While we did not find evidence that intrinsic motivation influenced VWM performance, intrinsic motivation was not manipulated in the current study. It would therefore be useful to test

for this result in a design where intrinsic motivation is experimentally manipulated.’

We have now changed the formulation of those sentences. Hopefully it is clearer now.

Minor points:
1. Abstract (line 22): There appears to be an additional space typo in the section …’experiment. These results…’

Fixed.

2. Introduction (lines 30-39): Is the difference in flexible resource allocation between slot models and continuous resource models of VWM so clear cut? Couldn’t items show differences in encoding strength between WM slots, indicating flexible resource allocation within the slot framework?

It is indeed very well possible to have flexible-resource models that allocate VWM resource in a slot-like manner (i.e. to a subset of the items). We tested those kinds of ‘hybrid’ models in an earlier paper (Van den Berg, Awh, Ma, 2014; Psych Rev) and found that they perform slightly better than resource-models without slots and much better than slot-models without flexible resources. Hence, there might be truth in both positions.

Nevertheless, proponents of slot-based models may have some problems with the statement that there can be “flexible resource allocation within the slot framework”. They often conceptualize working memory resource using terms that emphasize non-flexibility, such as “fixed slots”, “discrete slots” or “discrete chunks” (this is also nicely illustrated in the title of the seminal 2008 Nature paper by Zhang & Luck: “Discrete fixed-resolution representations in visual working memory”).

In any case, since the slot-vs-resource debate is largely tangential to the purposes of the present paper, we prefer to not open that can of worms in the introduction.

3. Procedure (line 115): The instruction ‘you will not receive any bonus if you get 0 points’ needs clarification in the manuscript. Does this mean that subjects will receive zero bonus payment for getting 0 points on any trial in the experiment (case a) or on one of the three trials randomly selected at the end to determine the bonus (case b)?

If case a, subjects’ motivation would plummet after receiving 0 points on a trial regardless of the reward group. It would therefore be important to exclude such subjects from the analyses and to

report how many subjects were excluded for this reason.

We have now clarified it that the correct interpretation was “case b” by adding more detail about what the subjects were exactly told about the bonus computation. We don’t have any reason to believe that they thought they would receive $0 bonus if on *any* trial they scored 0 points.

4. Figure 2 (line 149): It would be helpful to describe N in the figure legend. Presumably N refers to the set size.

Thanks for pointing this out. The other Reviewer had the same comment. We now write out “Set size” in the figure.

5. Results (lines 126 and 179): The three attention-checking questions presumably refer to the three points in the experiment where subjects need to press the space bar within four seconds. It would be useful to rephase this from attention-checking questions to space-bar attention checks or similar, so that readers do not confuse these with the attention checking quiz questions used at the end of the practice trials.

Yes, correct – but that was indeed not very clear. We clarified this now by referring to the space-bar-pressing “questions” as “catch trials” instead.

6. Results (lines 130 and 179): How many subjects were in each reward group after excluding subjects based on attention checks and response error distributions in each experiment?

We have now added this information.

7. Results (line 130): It would be useful to take the reader through how the circular variance of the response error distribution is calculated.

We added a footnote to explain this.

8. Results (line 179-201): The analyses looking at intrinsic motivation scores are interesting. The

authors tested whether reward condition was related to subjects' intrinsic motivation scores and intrinsic motivation sub-scales influenced VWM performance. It could also be interesting to look at whether intrinsic motivation scores influence VWM performance as a function of extrinsic reward conditions. Put another way, the authors could do the median split analyses in Figure 3B as a function of extrinsic reward condition. However, I will leave this up to the authors discretion, as it is not critical to their main research aims.

We are not sure what the Reviewer is suggesting here. We had 4 different extrinsic reward conditions ($0.50, $1, $2, $4) – but we already split by that, so that is probably not what the Reviewer meant. What the Reviewer perhaps meant instead, is to do a median split on the *actual* payout (within each condition). However, that would trivially result in finding an effect, because of the way that the payment structure was set up: the better a subject performed the more extrinsic reward they received.

In case we entirely missed the Reviewer's point, we'd be grateful for a clarification.

9. Appendix (line 348): The authors should state what the function for mapping error to reward in experiment 2 was.

We have added this information.
