# Supplementary material for: No effect of monetary reward in a visual working memory task
Source: PLoS One. 2023 Jan 17;18(1):e0280257. doi: 10.1371/journal.pone.0280257 (PMC9844926; doi:10.1371/journal.pone.0280257)
Supplement: Attachment — Submitted filename: rebuttal_20221212.docx [file pone.0280257.s003.docx]

**RESPONSE LETTER TO EDITOR AND REVIEWERS**

We thank the reviewers for their helpful comments and the Editor for his detailed explanation of the delay in processing our submission and his guidance with regard to how we should best deal with the remaining minor comments.

The main changes in the revised manuscript are as follows:

- We moved the simulation results from Appendix to Introduction. This is now Figure 1B. The original Figure 1B (a copy of the instruction screen) was removed, because most of that information can also be found in the main text.
- We extended the General Discussion with a discussion of the implication of our findings for the resource-rational model
- We extended the General Discussion with a summary of the seemingly mixed literature on the question of whether VWM performance and capacity can be increased through experimental manipulations. Our conclusion is that the literature might not be as mixed as it appears: the effects that have been reported previously (Bengson & Luck 2016; Brissenden et al. 2021) can be explained as effects happening at the *decision* stage, while no study thus far has delivered convincing evidence for an effect on VWM *capacity* (i.e., the encoding stage). In other words: while VWM performance is sensitive to certain experimental manipulations, VWM *capacity* does not seem to be so.

You can find detailed responses to all Reviewer comments below.

**EDITOR COMMENTS**

With regards to the previous round of reviews, I think you have clearly addressed all of the major concerns raised by the initial reviewers. The potentially outstanding point being Original Reviewer 2’s question about examining the effect of intrinsic motivation on VWM performance as a function of extrinsic motivation—however, I too was unable to decipher their question, and am happy to leave it at that.

Turning to the comments of the two new reviewers, you will see that their (relatively minor) criticisms overlap with regards to discussing some of the larger theoretical implications of your results. I share their view on this point. The general issue is that much of the Discussion is spent addressing potential study limitations and ruling out alternative accounts. Although there is value in the points you raise, there is relatively little in the way of linking your findings back to existing issues in the literature. For example, Reviewer 1 raises the question of whether your results constitute a falsification of the resource rational framework, or simply limits its scope. Reviewer 2 raises similar concerns along these lines in regards to how your results appear difficult to reconcile with the findings of Bengson and Luck (2016). Clearly, results in the literature are mixed on the matter of flexible allocation of attention at the task level. I do not think the issues must be resolved here—nor do I think they can be, given the current state of play—but some comment on summarizing motivational factors that are and are not implicated in improving VWM performance would be ideal. Reviewer 2 provides some suggestions that are relevant to this that should be addressed.

I also sympathize with Reviewer 1’s view that the theoretical led was buried in relation to resource rational models. I found that the early slots vs. resources was appropriate in contextualizing the genesis of the current work and related ideas, and am happy for this to be left as is. However, I do strongly agree with the reviewer that more could be done to communicate how the resource rational model necessarily predicts a dependency between motivation (and intuitively monetary incentive; though note Reviewer 2’s comments on Camerer and Hogarth’s, 1999, earlier review) and performance. Bringing the model prediction figure in from the appendix to the introduction would be useful in this regard.

Overall, I think it is clear that your manuscript meets the main requirements for publication in PLOS ONE. Before accepting the manuscript for publication though, I would like to see the points raised by the reviewers addressed in a (minor) revision of the manuscript. I am confident that all of the questions/concerns raised by the reviewers can be addressed textually, and so I anticipate being able to make a final decision on a revision without sending the manuscript out for review again.

Thanks for these positive remarks, the guidance in how to address the Reviewer’s comments, and the offer to make a decision relatively quickly.

**REVIEWER #1**

I think this work convincingly highlights important boundary conditions for resource allocation in visual short-term memory, and I recommend it for publication. I have a few very minor suggestions for changes.

1) Currently, the theoretical contribution of this paper seems a little bit buried. I suggest a few changes below. I think these revisions would be nice to have, but are not essential, and I leave implementing them to the discretion of the authors.

First, I don’t quiet see why the authors lead with the ‘slots’ versus ‘resources’ debate, which is dropped after the first paragraph and – as the authors note in their response to a reviewer – ‘is largely tangential’ and a ‘can of worms’.

I think one alternative is to take out this paragraph and place more focus on the ‘resource-rational’ framework of visual memory. As part of this revision, I think bringing the model predictions from Appendix 1 to the introduction would be helpful.

To bring the predictions of the resource-rational model more to the forefront, we have moved the prediction figure from the Appendix to the main text (the new Figure 1B).

After careful consideration, and in accordance with the view of the Editor, we have decided to keep the opening of the paper as it is (i.e., motivate the work in the context of the slots vs resources literature).

Also, if it is true that explicit (monetary) incentives don’t affect visual short-term memory resource allocation – is this a falsification of the resource-rational framework or can it be accommodated to capture these differences? Perhaps I missed it, but this does not seem to be explicitly discussed even though the framework is brought up in the introduction.

At a minimum, it seems like some discussion of these theoretical takeaways would be nice. Currently, the Discussion/Conclusion sections read more like a disjointed list that rules out alternative explanations. I think supplementing it with some higher-level interpretation of the results-- one that is contextualized within existing frameworks/models of visual short-term memory – would elevate the theoretical contribution of the article and make it feel more integrated and self-contained.

We thank the Reviewer also for these suggestions!

We added a brief discussion of the implications of our results for the resource-rational theory of VWM to the Discussion section:

*Our results limit the generality of the resource-rational theory of VWM, but they do not imply a full refutation. As we discussed in our original publication of that theory (van den Berg & Ma, 2018), resource-rational optimization can take place at multiple timescales. Therefore, it could be that VWM allocation is rational only at timescales different from the ones used in the experiments presented here. Moreover, while it is commonly assumed that monetary reward increases motivation, there are also studies suggesting that reward may have very little (Camerer, Hogarth, Booth, & Science, 1999) or even detrimental effects on motivation (Murayama, Matsumoto, Izuma, & Matsumoto, 2010) and cognitive control mechanisms (Yee & Braver, 2018). Therefore, it is possible that monetary reward is simply not an effective incentive when it comes to regulation of cognitive resources, even if those resources are handled in a rational manner in response to other experimental factors. Future studies could try to manipulate motivation through other kinds of incentives, such as coupling performance with curiosity fulfillment (FitzGibbon, Lau, & Murayama, 2020; Gottlieb, Lopes, & Oudeyer, 2016) or time penalties.*

We also added two paragraphs that together summarizes the current literature on the question to what extent VWM performance and capacity are sensitive to experimental manipulations. Our conclusion is that the literature might not be as mixed as it first appeared: the effects that have been found in previous literature (Bengson & Luck 2016; Brissenden et al. 2021) can be explained as effects happening at the *decision* stage, while no study thus far has delivered convincing evidence for an effect on VWM capacity (i.e., the encoding stage). In other words: while VWM performance is sensitive to certain experimental manipulations, VWM *capacity* does not seem to be so:

*The literature on whether total VWM capacity can be manipulated experimentally is small and has thus far produced results that seem to be mixed. Several of those studies have used paradigms similar to the one used here. A recent study by Brissenden et al. (2021) tested for effects of reward in an orientation recall task with a set size of two. One difference with our task is that one of the items in their experiment always had a higher probing probability than the other one. Subjects were informed about the probabilities by either a pre-cue (presented before the stimulus) or a retro-cue (presented after the stimulus). The authors found that reward had a beneficial effect on how subjects prioritized cued items over uncued ones, in the sense that rewards decreased recall error for cued items and increased recall error for non-cued item. However, consistent with the results presented here, they found no evidence for an effect of reward on total memory capacity. In another recent by ourselves (Mystakidou & van den Berg, 2020), we ‘gamified’ the continuous-recall paradigm as a way to manipulate subject motivation. While this manipulation affected both self-reported motivation scores and pupil sizes, also in that study we found that VWM performance was unaffected by any of the experimental manipulations. In a final study that used the orientation recall paradigm, Manga et al. (2020) tested for effects of monetary reward on memory precision in younger and older adults. One possibly important difference with the previous three studies is that they presented the stimuli sequentially and with a relatively longer duration (800 ms per stimulus, compared to 50 to 200 ms for the entire stimulus display in the other three studies). They found evidence for a positive effect of reward on memory precision in the younger adults. However, the effect size was small and it did not replicate in the group with older subjects.*

*Possibly the most convincing evidence that VWM performance can be manipulated experimentally comes from an earlier study by Bengson and Luck (2016). However, their results are difficult to compare directly with the other studies discussed here, because their experimental paradigm different in several important ways. First, they used color stimuli instead of orientations. Second, and more important, their experimental manipulation consisted of varying the instructions (“focus on a subset”, “focus on all items”, “simply do your best”) rather than monetary reward or motivation. Third, and equally important, they used a full-display change-detection task, which has a more complicated decision stage (Keshvari, van den Berg, & Ma, 2012) than a single-probe continuous-recall task. Hence, there are two ways in which their subjects may have improved their performance: by increasing VWM capacity or by using a better decision strategy. One possible explanation of their results – which would reconcile them with the seemingly contradicting results discussed above – is that subjects who were instructed to “focus on all items” may have improved their performance by using a better comparison strategy (between the first and second display) at the decision stage, rather than by increasing their VWM capacities.*

2) There are a few minor typos--there may be more, but I caught the following: 3rd sentence of discussion E3 “is was based on 9 randomly selected trials” should be “it was based on 9 randomly selected trials”; last sentence of article “but monetary reward does not may not be one of them” should be “but monetary reward may not be one of them”.

Fixed. Thanks!

**REVIEWER #2**

The submitted manuscript examines three experiments testing the relationship between visual memory performance in an online continuous report task. Overall, I think the paper is clear and admirably concise. I think the introduction of a third experiment with a within-subjects manipulation helps demonstrate the generality of the conclusion here.

I have one concern, which may necessitate a textual change, and two questions.

1. The discussion was too focused on limitations of the study.

This is partly a stylistic difference (I just feel that such a negative discussion is both a drag on the paper and a missed opportunity to draw theoretical links) and partly a sense that the paper did not fully engage with its theoretical context.

There are interesting questions raised by the current study when examined in the context of, say, the Bengson and Luck (2016) result. I don't, for instance, know how to reconcile the idea that participants can be instructed to pay attention to all of the stimuli and appear to perform better under those instructions, and yet not see any effect of monetary reward on performance. The Bengson and Luck result undermines a large part of the enterprise to take accuracy directly and estimate an invariant capacity coefficient, so I think this work is highly useful beyond the confines of the resource rational model.

We thank the Reviewer for raising this point, which is similar to the main point raised by Reviewer 1. In the revised manuscript, have substantially extended the Discussion (see responses to Reviewer 1), which now also provides a possible way to reconcile the Bengson & Luck (2016) results with the studies that have found negative evidence for an effect. In short: it seems that the performance improvement reported by Bengson & Luck was due to an effect at the *decision* stage of the task, not at the encoding stage. The more general conclusion we draw from our literature review is that while VWM performance can be manipulated experimentally (through the decision stage), VWM *capacity* seems to be immutable.

The next two points are ones that I think may also warrant consideration in the discussion, but it is up to the authors how deeply they wish to engage with them (or whether to engage with them at all).

2. Optimising performance over many trials may be distinct from optimising performance in a single trial.

While I appreciate the decision to use two forms of reward computation in the final experiment, both randomly selecting a subset to apportion a reward and summing very small rewards over a

large number of trials require the participant to be vigilant for the entire experiment. The strict resource rational model may predict that the total amount of mnemonic/attentional resource can

be flexibly allocated on any time scale, but a slightly more relaxed version of the model could suggest that this level of control may only be possible on smaller time scales. The literature

cited in the introduction, where precision increases for elements in a memory array that are rewarded more heavily than others, only implies that the distribution of performance is asymmetric not that the total amount changes (this is clear in the introduction), and that this occurs on a shorter time scale.

In attempting to reconcile these results with the current study there are three possibilities I can think of: 1. Participants can control both the resource amount and distribution, but only on shorter time scales; 2. Participants can control the distribution of resources but not the total amount; 3. Participants can control the amount of resources but monetary rewards may not have much of an effect.

The first possibility has a history in the literature on vigilance and arousal. The last option is possible given that increasing performance and motivation in participants through the use of

monetary rewards is fickle at best. Camerer and Hogarth's (1999; J. Risk and Uncertainty) review of monetary rewards in (mostly economics) experiments found many have no effect on performance, and some have a negative effect.

Thanks for sharing this interesting thought, which we have included in the revised Discussion.

3. What are the implications for models of VSTM performance that distinguish memory capacity from attentional control?

The primary implications drawn from the experiments focus on resource rational models of VSTM performance. Another class of VSTM theory that this bears upon directly are those that distinguish between transient attentional control and an upper bound on memory capacity (see, e.g., Adam, Mance, Fukuda, and Vogel, 2015; J. Cog. Neuro.). It seems difficult to reconcile that performance is bounded both attentional control and an invariant VSTM capacity limit, and that both are insensitive to any effect of monetary rewards.

We did not fully understand the point that the reviewer raised here. Since this point was presented as ‘optional’ by the reviewer and may partly be covered by the revised discussion about effects on VWM performance vs effects on VWM capacity, we assume that it is fine if we do not further engage with it.

Minor points

============

1. The variable precision model was estimated, but neither the predictions nor the MLE/MAP parameters were presented. Even though it's not the direct aim of the current study, it is useful to

present all of the details surrounding the fits even if just in an appendix.

We’ve inserted a table with ML estimates (Table 1 in the main text).

2. The cited Morey et al. (2011) result has antecedents in the work of Bonnel and Hafter (1998; Percept Psychophys.) and, prior to that, by Taylor, Lindsay, and Forbes (1967; Acta Psychologica).

Thanks! We added these references.
